# Supplementary material for: Parkinson’s Disease and Diabetes Mellitus: Individual and Combined Effects on Motor, Cognitive, and Psychosocial Functions
Source: Healthcare (Basel). 2023 May 4;11(9):1316. doi: 10.3390/healthcare11091316 (PMC10178005; doi:10.3390/healthcare11091316)
Supplement: Supplementary file 1 [file healthcare-11-01316-s001.zip › healthcare-2147601-supplementary.pdf]

Supplementary Table S1. Participant Performance on Motor, Motor-Cognitive, and Cognitive Assessments.

	HOA Total n=170		PD-Only Total n=162		DM-Only Total n=56		PD+DM Total n=36		df				Main effect of PD	Main effect of DM		PD X DM			Main effect of PD			Main effect of DM			PD X DM			Main effect of PD			Main effect of DM			PD X DM			Main effect of PD	Main effect of DM		PD X DM						Main effect of PD			Main effect of DM			PD X DM		
	n	Mean ± STD	n	Mean ± STD	n	Mean ± STD	n	Mean ± STD		F	p-value	η2	F	p-value	η2	F	p-value	η2	F	p-value	η2	F	p-value	η2	F	p-value	η2	F	p-value	η2	F	p-value	η2	F	p-value	η2	F	p-value	η2	F	p-value	η2	F	p-value	η2	F	p-value	η2	F	p-value	η2	F	p-value	η2
Preferred Forward Gait Speed (m/s)†	145	1.09 ± 0.2	140	1.01 ± 0.3	47	0.99 ± 0.3	26	0.97 ± 0.3	354	6.30	0.013	0.017	5.86	0.016	0.016	0.77	0.38	0.002	12.52	<0.001	0.032	4.74	0.03	0.012	0.94	0.332	0.002	9.62	0.002	0.025	4.28	0.039	0.011	0.94	0.332	0.002	**11.51**	**0.001**	**0.03**	3.50	0.062	0.009	0.91	0.34	0.002	3.421	0.065	0.009	3.796	0.052	0.01	0.688	0.407	0.002
Fast Forward Gait Speed (m/s)†	145	1.53 ± 0.4	140	1.39 ± 0.4	46	1.39 ± 0.4	26	1.36 ± 0.5	353	7.76	0.006	0.021	3.55	0.06	0.01	0.88	0.35	0.002	15.51	<0.001	0.04	2.59	0.109	0.007	1.11	0.293	0.003	19.59	<0.001	0.05	3.91	0.049	0.01	1.07	0.301	0.003	**17.04**	**<0.001**	**0.044**	**3.94**	**0.048**	**0.01**	1.14	0.286	0.003	**6.506**	**0.011**	**0.017**	**4.169**	**0.042**	**0.011**	0.951	0.33	0.002
Preferred Backward Gait Speed (m/s)†	145	0.77 ± 0.3	139	0.63 ± 0.3	46	0.71 ± 0.3	26	0.57 ± 0.3	352	18.69	<0.001	0.05	2.29	0.131	0.006	0.01	0.914	0	27.91	<0.001	0.072	1.66	0.199	0.004	0.00	0.984	0	31.46	<0.001	0.08	2.69	0.102	0.007	0.00	0.97	0	**25.80**	**<0.001**	**0.066**	2.64	0.105	0.007	0.02	0.878	0	**8.773**	**0.003**	**0.022**	2.973	0.086	0.008	0.101	0.751	0
Chair Stands	148	13.01 ± 5	140	10.98 ± 4.9	47	10.94 ± 5.1	27	10.41 ± 5.7	358	7.42	0.01	0.02	4.33	0.04	0.012	0.81	0.37	0.002	18.11	<0.001	0.046	4.06	0.045	0.01	1.32	0.251	0.003	16.39	<0.001	0.042	4.22	0.041	0.011	1.31	0.253	0.003	**18.53**	**<0.001**	**0.047**	3.49	0.062	0.009	1.30	0.255	0.003	**5.11**	**0.024**	**0.013**	**3.916**	**0.049**	**0.01**	0.942	0.332	0.002
6 MWT (m)†	144	422.93 ± 104.8	135	374.74 ± 102.1	47	366.69 ± 136.8	27	334.31 ± 132	349	14.38	<0.001	0.039	11.86	0.001	0.032	0.28	0.597	0.001	24.79	<0.001	0.062	10.24	0.001	0.026	0.36	0.547	0.001	21.44	<0.001	0.054	10.14	0.002	0.025	0.36	0.549	0.001	**18.11**	**<0.001**	**0.046**	**10.06**	**0.002**	**0.025**	0.34	0.562	0.001	**6.981**	**0.009**	**0.018**	**10.385**	**0.001**	**0.026**	0.236	0.628	0.001
One leg stand (s)	82	16.7 ± 11.5	131	15.97 ± 10.7	28	13.72 ± 11.6	24	11.96 ± 8.2	261	0.01	0.91	0	3.88	0.05	0.015	0.25	0.62	0.001	2.17	0.142	0.008	3.71	0.055	0.014	0.14	0.707	0.001	1.61	0.205	0.006	3.43	0.065	0.013	0.14	0.708	0.001	0.72	0.398	0.002	**4.75**	**0.030**	**0.016**	0.45	0.504	0.002	2.556	0.111	0.009	**5.264**	**0.023**	**0.018**	0.639	0.425	0.002
360 degree turn (steps)	66	3.02 ± 1.2	117	5.85 ± 5.7	18	4.5 ± 5.1	18	4.91 ± 2.7	215	49.68	<0.001	0.188	2.32	0.13	0.011	0.54	0.46	0.003	20.31	<0.001	0.085	0.00	0.971	0	2.71	0.102	0.011	17.17	<0.001	0.072	0.01	0.922	0	2.59	0.109	0.011	**12.85**	**<0.001**	**0.054**	0.01	0.926	0	2.58	0.11	0.011	**9.849**	**0.002**	**0.037**	0.27	0.604	0.001	0.939	0.334	0.004
360 degree turn (s)	66	6.21 ± 2.2	117	11.26 ± 6.4	17	6.59 ± 2.9	18	10.22 ± 4.1	214	59.64	<0.001	0.218	1.45	0.23	0.007	0.04	0.85	0	52.27	<0.001	0.196	0.25	0.616	0.001	0.88	0.349	0.003	32.78	<0.001	0.123	0.44	0.51	0.002	1.06	0.304	0.004	**23.74**	**<0.001**	**0.089**	0.46	0.498	0.002	1.29	0.257	0.005	**5.043**	**0.026**	**0.021**	0.048	0.827	0	2.183	0.141	0.009
TUG-Baseline (s)	158	8.79 ± 2.7	154	11.43 ± 6.8	49	10.6 ± 5	33	12.13 ± 6.5	390	16.44	<0.001	0.04	3.28	0.07	0.008	0.11	0.74	0	28.94	<0.001	0.067	3.39	0.066	0.008	0.85	0.357	0.002	26.21	<0.001	0.061	3.71	0.055	0.009	0.86	0.355	0.002	**20.49**	**<0.001**	**0.047**	**3.91**	**0.049**	**0.009**	0.65	0.422	0.001	**7.344**	**0.007**	**0.017**	**4.229**	**0.04**	**0.01**	0.484	0.487	0.001
TUG-Cognitive (s)	157	12.79 ± 5	153	16.21 ± 15.1	49	14.51 ± 6	32	15.83 ± 8.3	387	5.62	0.02	0.014	2.29	0.13	0.006	0.18	0.67	0	11.39	0.001	0.028	0.20	0.652	0.001	0.64	0.424	0.002	14.54	<0.001	0.036	0.70	0.405	0.002	0.68	0.411	0.002	**11.28**	**0.001**	**0.028**	0.78	0.377	0.002	0.60	0.44	0.001	1.596	0.207	0.004	1.108	0.293	0.003	0.316	0.574	0.001
TUG-Cognitive (Correct Subtractions Per Second)	157	0.3 ± 0.2	152	0.26 ± 0.3	49	0.26 ± 0.2	32	0.24 ± 0.3	386	2.89	0.09	0.007	1.79	0.18	0.005	0.38	0.54	0.001	2.95	0.087	0.008	0.96	0.329	0.002	0.05	0.826	0	6.54	0.011	0.017	2.20	0.139	0.006	0.06	0.805	0	**9.97**	**0.002**	**0.025**	1.52	0.218	0.004	0.12	0.731	0	**8.227**	**0.004**	**0.021**	1.336	0.249	0.003	0.192	0.661	0
FSST (s)	158	11.13 ± 5.1	134	12.15 ± 4.6	41	11.32 ± 3.7	30	12.57 ± 5.2	359	4.90	0.03	0.013	0.89	0.35	0.002	0.10	0.76	0	9.79	0.002	0.025	0.24	0.626	0.001	0.03	0.875	0	4.66	0.031	0.012	0.04	0.837	0	0.04	0.84	0	3.83	0.051	0.01	0.04	0.837	0	0.03	0.874	0	0.72	0.397	0.002	0.092	0.762	0	0.001	0.972	0
BPST (Product Score)	153	14.92 ± 8.9	156	17.87 ± 10.7	51	13.8 ± 9.8	35	15.14 ± 9	391	1.74	0.19	0.004	2.27	0.13	0.006	0.42	0.52	0.001	5.76	0.017	0.015	1.64	0.202	0.004	0.68	0.41	0.002	3.21	0.074	0.008	2.04	0.154	0.005	0.69	0.407	0.002	1.41	0.236	0.004	1.55	0.214	0.004	0.70	0.405	0.002	2.413	0.121	0.006	1.798	0.181	0.005	0.937	0.334	0.002
Serial 3 counting (% correct)	158	88.7 ± 18.9	155	93.46 ± 11	51	87.12 ± 20.8	34	83.54 ± 25.3	394	0.89	0.35	0.002	0.29	0.59	0.001	0.09	0.77	0	0.85	0.358	0.002	4.03	0.045	0.009	4.21	0.041	0.01	0.04	0.842	0	5.24	0.023	0.012	4.22	0.041	0.01	0.34	0.558	0.001	3.84	0.051	0.009	**4.46**	**0.035**	**0.01**	0.104	0.747	0	3.863	0.05	0.009	**4.597**	**0.033**	**0.01**
MoCA score	159	24.87 ± 3.9	143	25.34 ± 3.8	45	23.69 ± 3.1	34	24 ± 4.7	377	4.57	0.03	0.012	8.27	<0.001	0.021	0.24	0.62	0.001	0.04	0.851	0	5.37	0.021	0.013	0.01	0.941	0	1.70	0.193	0.004	3.07	0.081	0.007	0.00	0.97	0	0.33	0.569	0.001	2.15	0.143	0.005	0.00	0.995	0	0.288	0.592	0.001	2.175	0.141	0.005	0	0.986	0
Trails A (s)	159	39.77 ± 36.1	144	46.27 ± 44.7	45	39.88 ± 21.2	33	46.09 ± 30.2	369	3.01	0.08	0.008	0.76	0.39	0.002	0.04	0.85	0	5.77	0.017	0.014	0.10	0.757	0	0.01	0.935	0	2.14	0.144	0.005	0.40	0.528	0.001	0.03	0.875	0	1.23	0.268	0.003	0.30	0.587	0.001	0.02	0.89	0	0.501	0.479	0.001	0.073	0.787	0	0.031	0.86	0
Trails B (s)	159	100.47 ± 66.8	143	115.5 ± 87.4	45	113.14 ± 79.1	33	112.6 ± 92.8	368	0.09	0.77	0	0.54	0.46	0.001	1.12	0.29	0.003	5.11	0.024	0.013	0.16	0.688	0	0.91	0.341	0.002	1.18	0.277	0.003	0.00	0.965	0	1.12	0.29	0.003	1.00	0.317	0.002	0.01	0.93	0	1.01	0.315	0.003	0.969	0.326	0.002	0.048	0.826	0	0.441	0.507	0.001
Trails Difference (B-A)	159	61.15 ± 52	143	70.98 ± 67.3	45	73.26 ± 65.5	33	66.51 ± 70.1	368	0.01	0.91	0	0.62	0.43	0.002	1.39	0.24	0.004	2.45	0.118	0.006	0.30	0.586	0.001	1.50	0.222	0.004	0.58	0.447	0.001	0.06	0.814	0	1.68	0.196	0.004	0.56	0.455	0.001	0.03	0.873	0	1.52	0.218	0.004	0.635	0.426	0.002	0.16	0.69	0	0.939	0.333	0.002
CWIT Color Naming Scaled Score	98	10.34 ± 2.5	91	9.09 ± 3.4	28	9.11 ± 3.2	22	9.45 ± 3	235	2.61	0.11	0.011	1.01	0.32	0.004	3.26	0.07	0.014	6.74	0.01	0.027	1.25	0.265	0.005	3.14	0.078	0.013	0.92	0.339	0.004	0.46	0.5	0.002	4.16	0.042	0.016	1.36	0.245	0.005	0.30	0.584	0.001	**4.08**	**0.044**	**0.016**	0.717	0.398	0.003	0.744	0.389	0.003	2.772	0.097	0.011
CWIT Word Reading Scaled Score	98	10.33 ± 2.5	91	9.77 ± 3.2	28	8.86 ± 3.6	22	9.41 ± 2.7	235	0.36	0.55	0.002	6.80	0.01	0.028	0.54	0.46	0.002	1.57	0.211	0.006	4.54	0.034	0.018	1.80	0.181	0.007	0.00	0.975	0	3.13	0.078	0.012	2.39	0.123	0.009	0.86	0.354	0.003	2.15	0.144	0.008	2.48	0.117	0.009	0.083	0.773	0	2.671	0.104	0.01	1.872	0.173	0.007
CWIT Inhibition Scaled Score	98	11 ± 2.4	91	9.08 ± 3.9	27	8.56 ± 3.5	22	8.82 ± 3.7	234	4.00	0.05	0.017	5.77	0.02	0.024	2.43	0.12	0.01	14.04	<0.001	0.054	8.03	0.005	0.031	4.91	0.028	0.019	3.15	0.077	0.012	5.41	0.021	0.02	6.44	0.012	0.024	**4.54**	**0.034**	**0.017**	**4.57**	**0.034**	**0.017**	**6.49**	**0.012**	**0.024**	0.527	0.469	0.002	**6.731**	**0.01**	**0.024**	**4.358**	**0.038**	**0.015**
CWIT Inhibition/Switching Scaled Score	97	10.06 ± 3.2	89	8.76 ± 4.1	26	8.54 ± 3.1	21	8.95 ± 3.4	229	1.17	0.28	0.005	2.67	0.1	0.012	2.84	0.09	0.012	5.78	0.017	0.024	1.67	0.198	0.007	2.79	0.096	0.011	2.16	0.143	0.009	1.17	0.281	0.005	3.23	0.073	0.013	**6.91**	**0.009**	**0.027**	0.49	0.486	0.002	3.36	0.068	0.013	0.425	0.515	0.002	0.932	0.335	0.004	2.349	0.127	0.009

Tower Test-Achievement Score	112	10.1 ± 2.5	111	10.05 ± 3.4	32	9.25 ± 3.1	31	11.32 ± 2.5	282	0.908	0.34	0.003	0.019	0.89	0	3.921	0.05	0.014
Tower Test-Time per move (s/total moves)	112	9.4 ± 3.2	111	7.99 ± 4	32	7.66 ± 4.1	31	10.23 ± 4	282	1.05	0.31	0.004	0.102	0.75	0	14.919	<0.001	0.05
Tower Test-Mean first move (s)	112	9.67 ± 2.9	111	10.87 ± 3.5	32	9.94 ± 3.2	31	12.68 ± 2.6	282	17.863	<0.001	0.06	4.588	0.03	0.016	2.57	0.11	0.009
Brooks Spatial Task (% correct)†	68	60.85 ± 20.9	103	66.29 ± 19.6	28	58.86 ± 21.3	18	58.56 ± 20.8	213	2.24	0.136	0.01	1.72	0.192	0.008	0.69	0.408	0.003
Corsi block (Product Score)	134	22.87 ± 15.1	156	26.99 ± 16.2	41	19.51 ± 11.8	36	31.31 ± 22.2	363	5.52	0.02	0.015	0.48	0.49	0.001	1.27	0.26	0.003
SF12 (PCS)†	160	49.21 ± 9.7	136	41.52 ± 9.4	50	44.45 ± 11.6	32	38.85 ± 10.1	374	49.55	<0.001	0.115	9.95	0.002	0.023	0.68	0.41	0.002
SF12 (MCS)†	160	49.32 ± 8.9	136	45.8 ± 9.9	50	47.69 ± 10.4	32	42.89 ± 10.1	374	14.52	<0.001	0.037	3.23	0.073	0.008	0.28	0.601	0.001
Life Space Questionnaire	95	6.27 ± 1.1	50	6.66 ± 1.3	33	6.09 ± 1.5	11	5.91 ± 1	185	0.05	0.83	0	0.12	0.74	0.001	0.56	0.45	0.003
PASE	124	108.55 ± 68.9	136	105.72 ± 71.1	33	111.12 ± 78.7	34	89.31 ± 67.2	323	0.58	0.45	0.002	0.72	0.4	0.002	0.65	0.42	0.002
CPF	165	21.27 ± 4.1	159	19.19 ± 5	56	19.39 ± 5.5	35	18.37 ± 5.4	411	10.36	<0.001	0.025	0.92	0.34	0.002	0.03	0.86	0
BDI-II	124	5.99 ± 5.7	136	12.32 ± 7.7	33	10.03 ± 10.6	33	14.21 ± 10.9	322	35.52	<0.001	0.099	1.73	0.19	0.005	1.03	0.31	0.003
ABC	126	60.67 ± 28.4	159	68.85 ± 24.1	39	58.47 ± 31.3	35	59.85 ± 26.4	355	2.60	0.11	0.007	3.20	0.07	0.009	0.67	0.41	0.002
Falls in last year (#)	159	0.54 ± 2.02	162	11.15 ± 50.13	52	3.04 ± 12.84	36	2.75 ± 3.86	405	81.04	<0.001	0.167	1.77	0.18	0.004	7.86	0.01	0.019
Fear of falling§	166	1.98 ± 1.31	159	3.20 ± 1.65	55	2.30 ± 1.68	35	3.01 ± 1.20	411	49.66	<0.001	0.108	0.48	0.49	0.001	0.01	0.94	0
Quality of Life§	161	5.51 ± 1.22	157	5.10 ± 1.14	55	5.26 ± 1.30	34	5.16 ± 1.33	403	2.80	0.1	0.007	0.27	0.6	0.001	1.46	0.23	0.004

Tower Test-Achievement Score	0.704	0.402	0.002	0.624	0.43	0.002	6.265	0.013	0.021	0.59	0.443	0.002	0.624	0.43	0.002	6.296	0.013
Tower Test-Time per move (s/total moves)	1.995	0.159	0.007	0.379	0.539	0.001	13.586	<0.001	0.046	2.004	0.158	0.007	0.282	0.596	0.001	13.412	<0.001
Tower Test-Mean first move (s)	16.725	<0.001	0.055	5.429	0.021	0.018	2.647	0.105	0.009	13.528	<0.001	0.045	5.459	0.02	0.018	2.707	0.101
Brooks Spatial Task (% correct)†	0.41	0.523	0.002	0.69	0.409	0.003	0.80	0.372	0.003	0.19	0.661	0.001	0.74	0.39	0.003	0.83	0.363
Corsi block (Product Score)	6.82	0.009	0.017	0.25	0.618	0.001	3.64	0.057	0.009	2.66	0.104	0.007	0.04	0.835	0	3.37	0.067
SF12 (PCS)†	59.59	<0.001	0.133	8.59	0.004	0.019	0.53	0.467	0.001	45.11	<0.001	0.1	7.39	0.007	0.016	0.56	0.454
SF12 (MCS)†	13.11	<0.001	0.034	3.45	0.064	0.009	0.25	0.619	0.001	7.91	0.005	0.02	2.44	0.119	0.006	0.21	0.647
Life Space Questionnaire	0.40	0.526	0.002	1.95	0.164	0.01	1.47	0.228	0.007	0.33	0.565	0.002	1.88	0.172	0.01	1.46	0.229
PASE	1.40	0.237	0.004	0.49	0.486	0.001	0.81	0.368	0.002	3.06	0.081	0.009	1.21	0.271	0.004	0.86	0.355
CPF	22.86	<0.001	0.05	5.48	0.02	0.012	1.06	0.305	0.002	26.07	<0.001	0.057	7.16	0.008	0.016	1.03	0.311
BDI-II	50.22	<0.001	0.132	7.73	0.006	0.02	1.33	0.25	0.003	32.22	<0.001	0.083	4.66	0.032	0.012	1.34	0.248
ABC	4.06	0.045	0.011	2.38	0.124	0.006	0.71	0.4	0.002	1.95	0.163	0.005	3.32	0.069	0.009	0.66	0.417
Falls in last year (#)	7.90	0.005	0.019	0.44	0.506	0.001	1.97	0.161	0.005	7.81	0.005	0.019	0.28	0.596	0.001	1.94	0.165
Fear of falling§	60.59	<0.001	0.129	0.33	0.568	0.001	2.38	0.124	0.005	63.67	<0.001	0.134	1.00	0.318	0.002	2.31	0.129
Quality of Life§	12.69	<0.001	0.03	0.25	0.618	0.001	0.98	0.322	0.002	5.48	0.02	0.013	0.00	0.996	0	1.09	0.298

Tower Test-Achievement Score	0.021	0.129	0.719	0	0.903	0.343	0.003	**6.163**	**0.014**	**0.021**	**4.597**	**0.033**	**0.015**	0.301	0.584	0.001	**4.432**	**0.036**	**0.015**
Tower Test-Time per move (s/total moves)	0.045	3.053	0.082	0.01	0.507	0.477	0.002	**13.21**	**<0.001**	**0.044**	0.035	0.852	0	0.146	0.703	0	**11.166**	**0.001**	**0.037**
Tower Test-Mean first move (s)	0.009	**8.198**	**0.005**	**0.027**	**6.111**	**0.014**	**0.02**	3.156	0.077	0.01	**9.02**	**0.003**	**0.029**	**5.15**	**0.024**	**0.017**	2.577	0.11	0.008
Brooks Spatial Task (% correct)†	0.004	0.06	0.803	0	0.70	0.402	0.003	0.78	0.379	0.003	1.034	0.31	0.004	0.997	0.319	0.004	1.173	0.28	0.005
Corsi block (Product Score)	0.008	2.11	0.147	0.005	0.07	0.79	0	3.05	0.082	0.008	1.607	0.206	0.004	0.055	0.815	0	2.972	0.086	0.008
SF12 (PCS)†	0.001	**33.28**	**<0.001**	**0.074**	**7.69**	**0.006**	**0.017**	0.39	0.533	0.001	**12.03**	**0.001**	**0.027**	**8.334**	**0.004**	**0.018**	0.22	0.639	0
SF12 (MCS)†	0.001	**12.51**	**<0.001**	**0.032**	1.73	0.19	0.004	0.15	0.695	0	1.692	0.194	0.004	2.324	0.128	0.006	0.443	0.506	0.001
Life Space Questionnaire	0.007	0.22	0.641	0.001	1.46	0.229	0.007	1.49	0.223	0.008	0.311	0.578	0.002	1.513	0.22	0.008	1.702	0.194	0.009
PASE	0.003	1.13	0.29	0.003	1.41	0.235	0.004	1.20	0.275	0.004	1.148	0.285	0.003	1.247	0.265	0.004	1.087	0.298	0.003
CPF	0.002	**23.67**	**<0.001**	**0.052**	**6.78**	**0.01**	**0.015**	0.84	0.361	0.002	**3.897**	**0.049**	**0.008**	**8.05**	**0.005**	**0.017**	0.41	0.522	0.001
BDI-II	0.003	**29.52**	**<0.001**	**0.077**	**4.35**	**0.038**	**0.011**	1.21	0.273	0.003	**6.256**	**0.013**	**0.016**	**5.625**	**0.018**	**0.014**	0.655	0.419	0.002
ABC	0.002	0.74	0.391	0.002	2.59	0.108	0.007	0.75	0.388	0.002	2.842	0.093	0.008	3.151	0.077	0.008	1.123	0.29	0.003
Falls in last year (#)	0.005	**8.82**	**0.003**	**0.021**	0.32	0.572	0.001	2.37	0.125	0.006	2.12	0.146	0.005	0	0.992	0	0.853	0.356	0.002
Fear of falling§	0.005	**47.74**	**<0.001**	**0.1**	1.24	0.266	0.003	1.68	0.196	0.004	**6.357**	**0.012**	**0.013**	2.265	0.133	0.005	0.743	0.389	0.002
Quality of Life§	0.003	**9.43**	**0.002**	**0.021**	0.07	0.797	0	0.99	0.321	0.002	1.644	0.201	0.004	0.014	0.907	0	0.674	0.412	0.002

Numbers are bolded for significant p-values adjusted for education, sex, and race. † A parametric test was used for the unadjusted p-values. Abbrevations: MWT, Minute Walk Time; TUG, Timed Up and Go; FSST, Four Square Step Test; BPST, Body Position Spatial Test; MoCA, Montreal Cognitive Assessment; CWIT, Color Word Interference Test; SF12, Short Form 12; PASE, Physical Activity Scale for the Elderly; CPF, Compostiy Physical Function; BDI-II, Beck's Depression Inventory II; ABC, Activities-specific Balance Confidence Scale. P<0.05. Significant *p* and *eta* squared values are bolded.

Supplementary Table S2. Parkinson Medication State.

	OFF-PD Meds		ON-PD Meds	
	**n**	Mean ± STD	n	Mean ± STD
Preferred Backward Gait Speed (m/s)	83	0.58 ± 0.27	82	0.67 ± 0.30
360 degree turn (steps)	82	12.12 ± 7.19	53	9.57 ± 3.69
360 degree turn (s)	82	6.37 ± 6.46	53	4.72 ± 2.77
TUG-Cognitive (Correct Subtractions Per Second)	79	0.03 ± 0.04	105	0.43 ± 0.26
Corsi Product Score	82	24.70 ± 14.66	110	30.11 ± 19.11

Comparison to determine if medication state influenced the main effect of Parkinson's disease. Significant findings are presented.
